# Manifestaciones mucocutáneas y su relación con el recuento de linfocitos T CD4 en pacientes infectados con el virus de inmunodeficiencia humana hospitalizados en Medellín, Colombia

**DOI:** 10.7705/biomedica.6117

**Published:** 2022-06-01

**Authors:** Ana María Sanín, Ángela María Londoño, Verónica Gil, Ana María Mejía, Hernán Darío Aguirre, Elsa María Vásquez, Catalina Valencia, Carolina Cardona

**Affiliations:** 1 Universidad CES, Medellín, Colombia Universidad CES Universidad CES Medellín Colombia; 2 Programa de Dermatología, Universidad CES, Medellín, Colombia Universidad CES Universidad CES Medellín Colombia; 3 Universidad CES, Medellín, Colombia; Hospital General, Medellín, Colombia Universidad CES Universidad CES Medellín Colombia; 4 Hospital General de Medellín, Medellín, Colombia Hospital General de Medellín Medellín Colombia; 5 Universidad Pontificia Bolivariana, Medellín, Colombia Universidad Pontificia Bolivariana Universidad Pontificia Bolivariana Medellín Colombia

**Keywords:** HIV, síndrome de inmunodeficiencia adquirida, dermatología, infección, epidemiología, erupciones por medicamentos, hipersensibilidad a las drogas, inmunosupresión, HIV, acquired immunodeficiency syndrome, dermatology, infection, epidemiology, drug eruptions, drug hypersensitivity, immunosuppression

## Abstract

**Introducción.:**

Entre el 80 y el 95 % de los pacientes infectados por el virus de inmunodeficiencia humana (HIV) desarrollan manifestaciones en la piel que sirven como marcadores de su estado inmunológico.

**Objetivos.:**

Describir las manifestaciones dermatológicas y los factores clínicos y sociodemográficos de los pacientes hospitalizados con diagnóstico de HIV y su correlación con el recuento de linfocitos T CD4.

**Materiales y métodos.:**

Se hizo un estudio observacional de corte transversal y retrospectivo a partir del registro de las historias clínicas de 227 pacientes mayores de edad con diagnóstico de HIV, evaluados por dermatología en un hospital de Medellín, Colombia.

**Resultados.:**

Los 227 registros daban cuenta de 433 manifestaciones dermatológicas, el 64,4 % de ellas infecciosas. Las tres manifestaciones más frecuentes fueron candidiasis oral, condilomas acuminados y reacciones a medicamentos. Se encontró una relación estadísticamente significativa entre el virus del herpes zóster (HZ) diseminado y la sífilis secundaria, con un recuento de CD4 entre 200 y 499 células/mm^3^ (p=0,04 y 0,028, respectivamente), y entre la candidiasis oral y un recuento de CD4 menor de 100 células/ mm^3^ (p=0,008).

**Conclusiones.:**

La relación entre el herpes zóster diseminado y un recuento de CD4 entre 200 y 499 células/mm^3^ sugiere que, a pesar de los recuentos altos, se pueden presentar formas graves de la enfermedad debido a una posible disfunción de las células T y el agotamiento del sistema inmunológico. La relación entre la candidiasis oral y un recuento de CD4 menor de 100 células/mm^3^ plantea la posibilidad de considerar esta infección micótica como un marcador importante de debilitamiento inmunológico de los pacientes con HIV.

## Abstract (en)

**Introduction.:**

About 80-95% of patients infected with the human immunodeficiency virus (HIV) develop skin manifestations, which are markers of the patients’ immune status. Objective. To describe the dermatologic manifestations and the clinical and sociodemographic factors of hospitalized patients diagnosed with HIV and their correlation with CD4 T-lymphocyte count.

**Materials and methods.:**

We conducted an observational, cross-sectional, and retrospective study of the medical records of 227 adult patients with HIV diagnosis evaluated by dermatology in a hospital in Medellín, Colombia.

**Results.:**

We included 227 patient records with 433 dermatologic manifestations, 64.4% of them infectious. The most frequent manifestations were oral candidiasis, condylomata acuminata, and drug reactions. Moreover, a statistically significant relationship was found between disseminated herpes zoster virus and secondary syphilis with a CD4 count between 200-499 cells/mm^3^ (p=0.04 and 0.028, respectively). There was also a statistically significant relationship between oral candidiasis and a CD4 count of less than 100 cells/ mm^3^ (p=0.008).

**Conclusions.:**

The relationship between disseminated herpes zoster with CD4 between 200-499 cells/mm^3^ suggests that, despite having high CD4 counts, severe forms of the disease may occur due to possible T-cell dysfunction and depletion of the immune system. Additionally, the relationship between oral candidiasis and CD4 less than 100 cells/mm^3^ indicates the potential role of oral candidiasis as an essential marker of weakened immune status in HIV patients.

La piel es el órgano más visible y extenso del ser humano y en ella se pueden evidenciar manifestaciones de enfermedades sistémicas, como sucede con la infección por el virus de la inmunodeficiencia humana (HIV). Entre el 80 y el 95 % de los pacientes infectados por HIV desarrollan manifestaciones en la piel que no solo ocurren por disminución en el recuento de linfocitos T CD4, sino también, por cambios en el perfil de expresión de citocinas hacia linfocitos Th2 (T *helper* 2), por mimetismo molecular y por expresión de superantígenos ^1^^,^^2^.

Las manifestaciones dermatológicas en los pacientes con HIV se caracterizan por su presentación atípica, su mayor gravedad y su resistencia al tratamiento. Muchas de ellas hacen parte de las manifestaciones clínicas definitorias del síndrome de inmunodeficiencia adquirida (sida) y, además de ofrecer pistas para el diagnóstico de esta infección, también sirven como marcador del estado inmunitario de los pacientes, de la progresión de la infección y de la posible presencia de enfermedades sistémicas asociadas ^3^. A medida que el recuento de CD4 disminuye, el sistema inmunitario se deteriora, lo cual favorece un aumento en la incidencia y la gravedad de estas manifestaciones, las cuales pueden presentarse en cualquier momento de la enfermedad y frecuentemente son su manifestación inicial, lo que lleva a que los especialistas en dermatología sean quienes, usualmente, tienen el primer contacto con estos pacientes ^1^^,^^4^^,^^5^.

Dichas manifestaciones se pueden clasificar en dermatosis no infecciosas e infecciosas. Las infecciosas se clasifican según su etiología como bacterianas, virales, parasitarias o por hongos, en tanto que las no infecciosas comprenden las dermatosis inflamatorias y las neoplasias ^1^^-^^3^^,^^6^. El diagnóstico de las enfermedades cutáneas en estos pacientes constituye un reto, ya que muchas se manifiestan con una gran variabilidad clínica debido al estado de inmunosupresión, por lo que muchas veces el diagnóstico requiere de la valoración de especialistas en dermatología y de biopsia ^5^.

En estudios realizados en los países de altos ingresos, se ha evidenciado el aumento de las hospitalizaciones y de la mortalidad debida a enfermedades crónicas y neoplasias no atribuibles al sida. En los países en desarrollo, sin embargo, se ha descrito que las enfermedades definitorias de sida aún son la principal causa de hospitalización y mortalidad; en Colombia, además, ha aumentado el número de nuevos casos, especialmente en grupos de alta vulnerabilidad, así como las limitaciones en el acceso a la terapia antirretroviral (TAR) ^4^.

En este sentido, los dermatólogos tienen un papel importante en el manejo integral de estos pacientes. En nuestro medio hay pocos estudios sobre las manifestaciones dermatológicas en pacientes con HIV, especialmente en el contexto hospitalario, por lo que el objetivo del presente estudio fue describir dichas manifestaciones y los factores clínicos y sociodemográficos de pacientes hospitalizados con diagnóstico de HIV y su correlación con el recuento de linfocitos T CD4.

## Materiales y métodos

Se hizo un estudio observacional de corte transversal e intención analítica a partir de una fuente secundaria. Se incluyeron las historias clínicas registradas entre agosto del 2014 y agosto del 2019 de pacientes mayores de 18 años con diagnóstico de HIV conocido o nuevo, hospitalizados o remitidos por dermatología, en un hospital público de tercer nivel de Medellín (Colombia) que opera como centro de remisión regional. Al ser un hospital público, un porcentaje significativo de los pacientes está afiliado al régimen subsidiado de salud y, además, es un centro de referencia para pacientes habitantes de calle y privados de la libertad. Se consideró como hospitalización por causa dermatológica la de aquellos pacientes remitidos desde la consulta dermatológica. Se excluyeron los pacientes menores de 18 años y aquellos sin registro de más del 20 % de los datos de las variables sociodemográficas y clínicas.

La información se recolectó entre agosto del 2019 y agosto del 2020 a partir de los registros de historias clínica del hospital después de una prueba piloto. Los pacientes habían estado hospitalizados en varias ocasiones durante el periodo de seguimiento, pero solo se recogió la información de la primera hospitalización. Se incluyeron variables sociodemográficas (cuadro 1) y clínicas, y se agruparon en manifestaciones dermatológicas infecciosas (virales, bacterianas, parasitarias o micóticas), y en no infecciosas (inflamatorias o tumorales). También, se tuvieron en cuenta variables como las comorbilidades y complicaciones relacionadas con la hospitalización. Se obtuvo información del recuento de linfocitos T CD4 y se agrupó según la clasificación de los *Centers for Disease Control and Prevention* (CDC) de Atlanta de 1993 en: >500 células/mm^3^, 200-499 células/mm^3^ y <200 células/ mm^3^; además, se incluyó un subgrupo con CD4 menor de 100 células/mm^3^.

 No se estableció un tamaño de muestra porque se trabajó con el censo de registros de los pacientes. El riesgo de sesgo de selección se consideró bajo, pues se controló con la inclusión del universo, así como el de información, ya que se excluyeron aquellas historias clínicas con más del 20 % de pérdida de datos sobre las variables sociodemográficas o clínicas. Los resultados de las variables cualitativas se presentaron mediante tablas de frecuencia y porcentajes y los de las variables cuantitativas, con tablas de promedios y desviación estándar en caso de distribución normal, o de medianas y rangos intercuartílicos en caso de distribución no normal. Se hicieron análisis estadísticos de las variables cualitativas con la prueba de ji al cuadrado y, de las variables cuantitativas, con las pruebas T de Student y U de Mann-Whitney. Los valores de p<0,05 se consideraron estadísticamente significativos. Los datos se procesaron utilizando el programa estadístico SPSS™, versión 23, con licencia de la Universidad CES.

### Consideraciones éticas

Se obtuvo la aprobación del Comité de investigación y de ética de la Universidad CES y del Hospital General de Medellín.

## Resultados

Se encontraron los registros de 240 pacientes y se seleccionaron 227 después de aplicar los criterios de exclusión. La edad media de los pacientes era de 37,36 años, con una desviación estándar (DE) de 11,712; el 81,5 % de los pacientes eran de sexo masculino, la mayoría afiliados al régimen subsidiado de salud (74,9 %); el 69,2 % correspondía a personas solteras y el 41,4 % se encontraba desempleado. El 3,5 % de esta población era mujer gestante, el 5,3 %, habitante de calle, y el 6,2 % se encontraba privado de la libertad. La gran mayoría de los pacientes (79,3 %) conocía el diagnóstico antes de la primera hospitalización y el 27,3 % ingresó por un diagnóstico dermatológico; el promedio de estancia hospitalaria fue de 24,71 días (cuadro 1).

**Cuadro 1:** Características sociodemográficas (N=227)

Características		Frecuencia (n)	Porcentaje (%)
Edad media: 37,36 años (18-75); DE=11,712			
Días de hospitalización: 24,71 (1-110); DE=19,14			
Sexo			
	Mujer	42	18,5
	Hombre	185	81,5
Régimen de salud			
	Contributivo	44	19,4
	Subsidiado	170	74,9
	Especial	13	5,7
Estado civil			
	Soltero	157	69,2
	Casado	18	7,9
	Unión libre	34	15
	Separado	10	4,4
	Viudo	3	1,3
Ocupación			
	Con trabajo	81	35,7
	Sin trabajo	94	41,4
	Estudiante	7	3,1
Extranjeros			
	Total	9	4%
	Venezuela	6	2,6
Poblaciones especiales			
	Gestantes	8	3,5
	Habitantes de calle	12	5,3
	Privados de la libertad	14	6,2
Diagnóstico de HIV			79,3
	Conocido previo al ingreso	180	20,7
	Desconocido previo al ingreso	47	27,3
	Hospitalización por diagnóstico dermatológico	62	

En los 227 registros seleccionados, se encontraron 433 manifestaciones mucocutáneas, con un promedio de 2,02 ± 1,18 por paciente. Las principales fueron la candidiasis oral y los condilomas acuminados, con el 22 % cada una, seguidas de las toxicodermias, con el 19,3 %, siendo más frecuentes las de tipo exantemático, con el 66 %, seguidas del síndrome de reacción a fármacos con eosinofilia y síntomas sistémicos (*Drug Reaction with Eosinophilia and Systemic Symptoms,* DRESS), con el 15,9 % (cuadro 2). Los medicamentos más relacionados con las toxicodermias fueron las sulfas (cuadro 3). La frecuencia de la dermatitis seborreica fue del 17,6 % y, las de la erupción papular pruriginosa del HIV, el herpes simple genital y el herpes zóster, del 11, 11,9 y 11,5 %, respectivamente; la del sarcoma de Kaposi fue del 10,6 % y la del herpes simple cutáneo fue del 9,7 %. En el cuadro 4 se presenta de forma detallada la frecuencia de los diagnósticos dermatológicos encontrados y su respectiva correlación con el recuento de CD4.

**Cuadro 2:** Distribución de las reacciones a medicamentos

Toxicodermia	%
Exantemática	63,6
DRESS	15,9
Erupción fija por medicamentos	9
Urticaria	9
Eritema multiforme	2,2

**Cuadro 3:** Principales grupos de medicamentos relacionados con las toxicodermias

Grupo de medicamento	Frecuencia (%)
Sulfas	40,9
Antibióticos diferentes a las sulfas	18
Antirretrovirales	13,6
Antiinflamatorios no esteroideos AINE	11,3
Antituberculosos	6,8
Anticonvulsivos	4,5

**Cuadro 4:** Prevalencia de manifestaciones dermatológicas en pacientes con diagnóstico de HIV y su relación con el recuento de linfocitos T CD4

Manifestación dermatológica	CD4 n (%)									p
	n (%)	<100		100-199		200-499		>500		
Inflamatorias	125	n	%	n	%	n	%	n	%	
Síndrome retroviral agudo	1 (0,4)	0	0	0	0	1	0,4	0	0	0,278
Síndrome de reconstitución inmunológica	3 (1,3)	3	2,4	0	0	0	0	0	0	0,479
Toxicodermias	44 (19,3)	30	24	4	11,4	7	14,9	3	15	15 0,264
Dermatitis seborreica	40 (17,6)	25	20	9	25,7	3	6,4	3	15	0,1
Dermatitis atópica	3 (1,3)	3	2,4	0	0	0	0	0	0	0,479
Psoriasis	2 (0,9)	1	0,8	0	0	1	2,1	0	0	0,721
Foliculitis eosinofílica	4 (1,8)	1	0,8	2	5,7	0	0	1	5	0,118
Erupción papular pruriginosa del HIV	25 (11,1)	10	8,1	5	14,3	9	19,1	1	5	0,146
Prúrigo nodular	3 (1,3)	2	1,6	0	0	1	2,1	0	0	0,79
Tumorales	29									
Sarcoma de Kaposi*	24 (10,7)	15	12,2	4	11,4	4	8,5	1	5	0,747
Cáncer de piel no melanoma	4 (1,8)	2	1,6	2	5,7	0	0	0	0	0,223
Neoplasia intraepitelial anal	1 (0,4)	0	0	0	0	1	2,1	0	0	0, 278
Infecciosas virales*	156									
Herpes simple cutáneo y de mucosa oral	22 (9,7)	13	10,4	4	11,4	3	6,4	2	10	0,854
Herpes simple genital	27 (11,9)	16	12,8	4	11,4	5	10,6	2	10	0,97
Herpes zóster	26 (11,5)	11	8,8	5	14,3	7	14,9	3	15	0,585
Herpes zóster diseminado	6 (2,6)	2	1,6	0	0	4	8,5	0	0	0,04
Molusco contagioso	16 (7)	8	6,4	4	11,4	3	6,4	1	5	0,737
Leucoplasia vellosa oral	4 (1,8)	2	1,6	2	5,7	0	0	0	0	0,223
Verruga vulgar	5 (2,2)	4	3,2	1	2,9	0	0	0	0	0,541
Condiloma acuminado	50 (22)	30	24	8	22,9	9	19,1	3	15	0,777
Infecciosas, bacterianas	34									
Piodermitis	16 (7)	6	5,6	2	5,7	6	12,8	1	5	0,396
Angiomatosis bacilar	2 (0,9)	1	0,8	0	0	1	2,1	0	0	0,723
Tuberculosis cutánea	1 (0,4)	0	0	0	0	1	2,1	0	0	0,278
Sífilis primaria	7 (3,1)	3	2,4	2	5,7	2	4,3	0	0	0,602
Sífilis secundaria	8 (3,5)	2	1,6	1	2,9	5	10,6	0	0	0,028
Infecciosas, micóticas	82									
Candidiasis oral	50 (22,1)	37	29,6	7	20	6	12,8	0	0	0,008
Intertrigo por *Candida*	4 (1,8)	2	1,6	1	2,9	1	2,1	0	0	0,883
Dermatofitosis	12 (5,3)	8	6,4	0	0	2	4,3	2	10	0,355
Criptocococosis cutánea	3 (1,3)	3	2,4	0	0	0	0	0	0	0,479
Histoplasmosis cutánea	10 (4,4)	9	7,2	0	0	1	2,1	0	0	0,143
Pitiriasis versicolor	3 (1,3)	1	0,8	1	2,9	1	2,1	0	0	0,707
Infecciosas, parasitarias	7									
Escabiosis	3 (1,3)	0	0	1	2,9	1	2,1	1	5	0,202
Pediculosis	1 (0,4)	0	0	0	0	1	2,1	0	0	0,278
Leishmaniasis	3 (1,3)	3	2,4	0	0	0	0	0	0	0,479
Total	433	253		69		84		24		

RIC: rango intercuartil

En cuanto al recuento de linfocitos T CD4, se encontró que el 70,5 % de los pacientes tenía el recuento de CD4 por debajo de 200 células/mm^3^ y, de estos, el 55,1 % lo tenía por debajo de 100 células/mm^3^ (cuadro 5).

**Cuadro 5:** Recuento de linfocitos T CD4

Recuento de linfocitos T CD4	n	%
Menor de 100	125	55,1
100 a 199	35	15,4
200 a 499	47	20,7
Mayor de 500	20	8,8

 Al hacer el análisis por subgrupos de manifestaciones, se encontró lo siguiente.

*Manifestaciones clínicas infecciosas*. La mayoría de las manifestaciones clínicas dermatológicas fueron las infecciosas, con 279 (64,4 %), clasificadas según su origen en virales, bacterianas, micóticas o parasitarias.

Virales. De las 279 manifestaciones clínicas infecciosas, 156 (55,9 %) fueron de etiología viral. Las principales manifestaciones fueron condilomas acuminados (50 casos), herpes simple (49 casos) y de 32 casos de herpes zóster, el 18,7 % fue de tipo diseminado, en tanto que hubo 19 casos de molusco contagioso. La única manifestación viral con una diferencia estadísticamente significativa frente al recuento de CD4 fue el herpes zóster diseminado, siendo más frecuentes los recuentos entre las 200 y las 499 células/mm^3^ (p=0,04). El 60 % de los condilomas acuminados se presentó con recuentos de CD4 menores de 100 células/mm^3^, pero sin una diferencia estadísticamente significativa.

Bacterianas. De las 279 manifestaciones clínicas infecciosas, 34 (12,2 %) fueron bacterianas y la piodermitis fue la más frecuente (16 casos). Se encontró una relación estadísticamente significativa entre un recuento de CD4 de 200499 células/mm^3^ y la sífilis secundaria (p=0,028).

Micóticas. Hubo 29,4 % (82 de 279) de infecciones micóticas y la más prevalente (50 casos) fue la candidiasis oral, seguida de la dermatofitosis (12 casos) y la histoplasmosis cutánea (10 casos). Se encontró una relación estadísticamente significativa entre la candidiasis oral y un recuento de CD4 menor a 100 células/mm^3^ (p=0,008). A pesar de que el 90 % de los pacientes con histoplasmosis cutánea y el 100 % de aquellos con criptococosis cutánea tenían recuentos de CD4 menores de 100 células/mm^3^, no se encontró una relación estadísticamente significativa con dicho valor.

Parasitarias. El 2,5 % de las manifestaciones infecciosas correspondió a parasitosis, con tres casos de escabiosis y tres casos de leishmaniasis. En todos los casos de leishmaniasis, los recuentos de CD4 fueron menores de 100 células/mm^3^, pero sin una relación estadísticamente significativa. Cabe resaltar que dos de los pacientes con *leishmaniasis* tenían también infección por *Histoplasma* spp. y uno de ellos presentaba coinfección de *Leishmania* spp. e *Histoplasma* spp. en piel, demostrada por PCR del tejido.

*Manifestaciones clínicas no infecciosas*. El 35,6 % (154 de 433) de las manifestaciones correspondía a no infecciosas, clasificadas en inflamatorias o tumorales, como sigue.

Inflamatorias. Se encontraron 125 manifestaciones inflamatorias, siendo las más comunes las toxicodermias, con el 19,3 % (n=44). El 77,3 % de las reacciones a medicamentos se presentaron con recuentos de CD4 menores de 200 células/mm^3^. La dermatitis seborreica se registró en 40 casos, el 85 % de ellos con recuentos de CD4 menores de 200 células/mm^3^. La erupción papular pruriginosa del HIV se diagnosticó en 25 casos, el 60 % de ellos con recuentos de CD4 menores de 200 células/mm^3^. Los tres casos de síndrome de reconstitución inmunológica se presentaron con recuentos de CD4 menores de 100 células/mm^3^. No hubo diferencias estadísticamente significativas entre estas manifestaciones inflamatorias y el recuento de linfocitos T CD4.

*Tumorales.* Se encontraron 29 manifestaciones tumorales, siendo la más frecuente el sarcoma de Kaposi, con 24 casos, el 79 % de los cuales registraba recuentos de CD4 menores de 200 células/mm^3^, sin una diferencia estadísticamente significativa.

### Comorbilidades

Se encontró que el 6,2 % de los pacientes eran hipertensos, el 6,2 % tenía una neoplasia hematológica asociada y el 4,4 % una neoplasia, y el 1,8 % era diabético. Entre las comorbilidades psiquiátricas, el 9,3 % correspondía a depresióel n, 7,9 % a trastorno de ajuste, el 0,9 % a trastorno afectivo bipolar y el 0,4 % a esquizofrenia.

### Complicaciones relacionadas con la hospitalización

Se encontró que el 9,3 % de los pacientes requirió manejo en la unidad de cuidados especiales, el 9,3 % en la unidad de cuidados intensivos y el 12,8 % murió durante la hospitalización.

## Discusión

En el presente estudio, se encontró que la mayoría de los pacientes con diagnóstico de HIV evaluados por dermatología, presentaban más de una manifestación dermatológica, con un promedio de 2,02 ± 1,18 manifestaciones por paciente, y las manifestaciones infecciosas fueron las más comunes. Las tres manifestaciones dermatológicas más frecuentes fueron la candidiasis oral, los condilomas acuminados y las reacciones a medicamentos.

Es importante señalar que el 70,5 % de los pacientes tenía un recuento de CD4 por debajo de 200 células/mm^3^ y que, en el 55,1 % de ellos, estaba por debajo de 100 células/mm^3^. Además, se estableció una relación entre el herpes zóster diseminado y la sífilis secundaria y un recuento de linfocitos T CD4 entre 200 y 499 células/mm^3^, y entre la candidiasis oral y un recuento menor de 100 células/mm^3^, lo que reitera que estas manifestaciones orientan el diagnóstico de HIV y, además, permiten conocer el estado inmunológico de los pacientes. Resalta, asimismo, el papel tan importante de los dermatólogos en la epidemia del VIH.

En un estudio de Álvarez, *et al*. ^7^, se describen las características clínicas y causas de hospitalización de 551 pacientes colombianos con diagnóstico de HIV con una edad media de 37 años, igual a la de nuestro estudio y similar a otro sobre manifestaciones dermatológicas del HIV en Medellín realizado por Gaviria, et al. ^8^, en el cual reportaron una edad promedio de 38 años. Esto coincide también con los hallazgos del informe de cuenta de alto costo de HIV del 2019 en el país, en el que se da cuenta del predominio en hombres con edades entre los 30 y los 34 años ^9^. Tanto en nuestro estudio como en el de Gaviria, *et al*. ^8^, se encontró que la mayoría de los pacientes era de sexo masculino: 87,1 % y 81,5 %, respectivamente. En el estudio de Álvarez, et al., más del 50 % de los pacientes tenía recuentos de CD4 menores de 200 células/ mm^3^ en el momento de la admisión y, en el nuestro, la mayoría (70,5 %) tenía estos mismos recuentos (200 células/mm^3^) en el primer ingreso hospitalario. Estos autores registraron que el 78 % de los pacientes tenía un diagnóstico conocido de HIV, similar a nuestro hallazgo de 79,3 % ^7^.

Titou, *et al*., reportaron en su estudio un promedio de dos manifestaciones dermatológicas por paciente, con un rango de 0 a 7, lo que concuerda con nuestro hallazgo de 2,02 manifestaciones dermatológicas en promedio por paciente ^10^. Pocos son los estudios locales que han descrito las manifestaciones dermatológicas en pacientes con HIV. En el estudio publicado por Gaviria, et al., se reportaron como las principales la infección por el virus del papiloma humano (PVH) (37,8 %), la infección por el virus de herpes simple (VHS) (23,2 %), la candidiasis oral (18,9 %), el sarcoma de Kaposi (4 %), la sífilis secundaria (0,57 %), la dermatitis seborreica (17,1 %), y las toxicodermias (5,15 %); los medicamentos más relacionados (55 %) fueron los antimicrobianos (sulfas, penicilinas y antituberculosos), seguidos de los antirretrovirales (22,2 %) ^8^. En el presente estudio, hubo hallazgos similares en cuanto a la candidiasis oral y los condilomas acuminados, con el 22 % cada una, y la dermatitis seborreica, con el 17,6 %. Sin embargo, se encontró un mayor porcentaje de toxicodermias, posiblemente porque este estudio se llevó a cabo en pacientes hospitalizados y, el de Gaviria, *et al*., en un contexto ambulatorio.

En nuestros casos, las reacciones a medicamentos se presentaron en el 19,3 % y fueron más frecuentes las de tipo exantemático (66 %), seguidas del DRESS (15,9 %); los medicamentos más implicados fueron las sulfas. Cabe resaltar que el 77,3 % de las toxicodermias se presentó con recuentos de CD4 menores de 200 células/mm^3^, aunque sin una relación estadísticamente significativa. Las reacciones a medicamentos se reportan con mayor frecuencia en pacientes infectados con HIV, especialmente, reacciones de hipersensibilidad retardada y ampollosas graves ^11^, cuyo riesgo aumenta con recuentos de CD4 entre 100 y 400 células/mm^3 (^^12^^,^^13^, probablemente por la disminución de linfocitos T reguladores en la piel ^13^.

Los principales medicamentos reportados en los estudios son trimetoprim-sulfametoxazol, aminopenicilinas, dapsona, abacavir, nevirapina y antiepilépticos. Se ha reportado que los pacientes con HIV tienen 10 a 50 veces más riesgo de presentar una toxicodermia por sulfametoxazol, dato que concuerda con nuestros resultados de 40,9 % de toxicodermias relacionadas con las sulfas. En el estudio de Coopman, *et al*. ^12^, el 30,2 % de los ingresos hospitalarios se atribuyó a toxicodermias, y las reacciones más frecuentes fueron las morbiliformes (74 %), lo que coincide con nuestros hallazgos de 19,3 %; las reacciones exantemáticas también fueron las más comunes (63,6 %), lo cual indica que a pesar de que estos pacientes tienen más riesgo de presentar reacciones ampollosas graves como el síndrome de Steven Johnson o la necrólisis epidérmica tóxica, las más frecuentes siguen siendo las exantemáticas o morbiliformes leves. En ambos estudios, los principales medicamentos relacionados fueron los antibióticos, especialmente los derivados de las sulfas, con el 50 % en el estudio de Coopman, *et al*., y el 40,9 % en el nuestro.

En cuanto a la candidiasis oral, esta se presentó en el 29,3 % de los casos, siendo una de las manifestaciones dermatológicas más frecuentes. Asimismo, se encontró una relación estadísticamente significativa entre la candidiasis oral y un recuento de CD4 menor de 100 células/mm^3^ (p=0,008). En la revisión bibliográfica, se encontraron datos similares ^10^^,^^14^^-^^16^: en un estudio sobre manifestaciones dermatológicas de pacientes con HIV y su relación con los CD4 en Turquía, se reportó que la candidiasis oral fue el principal diagnóstico, con un 12,4 %, así como una relación estadísticamente significativa entre esta y un recuento de CD4 menor de 200 células/mm^3^ (p=0,0001) ^17^. En otro estudio similar en Tailandia, se reportó la presencia de candidiasis oral en el 54,17 % de los pacientes, el 94,5 % de ellos con CD4 menores de 200 células/mm^3 (^^18^. Fernandes, *et al*. reportaron la presencia de candidiasis oral en el 21 % y, aunque no encontraron una diferencia estadísticamente significativa con el recuento de CD4, evidenciaron que la incidencia de candidiasis se incrementaba a medida que el recuento de CD4 disminuía ^19^. En nuestro estudio, también se registró la candidiasis oral como marcador de un estado inmunológico deficiente ^20^.

Con relación al herpes zóster, se ha descrito que los pacientes inmunocomprometidos tienen 20 veces más riesgo de contraerlo y que su incidencia aumenta a medida que disminuyen los linfocitos T CD4; además, las manifestaciones clínicas son graves y atípicas en ellos ^21^^-^^24^. En nuestro estudio, el herpes zóster se presentó en el 11,5 % de los pacientes y, el de tipo diseminado, en el 2,6 %; llama la atención que cuatro de los seis pacientes que presentaban formas diseminadas tenían recuentos de CD4 entre 200 y 499 células/mm^3^, con una relación estadísticamente significativa (p=0,04). Esto concuerda con un estudio en el que se reportaron tres pacientes con herpes zóster multidermatómico, recuentos altos de CD4 y cargas virales bajas, por lo que se planteó que, a pesar de la relación reportada entre herpes diseminado y disminución de la inmunidad celular, es posible que este se presente en el contexto de recuentos de CD4 altos y cargas virales suprimidas debido a agotamiento del sistema inmunológico y disfunción de las células T, a pesar de un buen control virológico ^24^.

La relación entre la leishmaniasis y el HIV es compleja y la incidencia de la coinfección ha ido aumentando con los años ^25^. Se ha descrito una modulación recíproca entre ambos patógenos, con un predominio en la reacción inmunitaria de los TH2 y de tipo humoral que resulta en la propensión a presentar ambas infecciones. En las personas con HIV, se inhibe la producción de interferón gamma, lo que altera la actividad lítica de los macrófagos y limita su capacidad para eliminar los amastigotes. Por otro lado, la leishmaniasis produce una desregulación de la reacción inmunitaria del huésped, lo cual genera una activación crónica que favorece la progresión del HIV ^26^. Clínicamente, la leishmaniasis en pacientes infectados con HIV se presenta con formas atípicas, difusas, incluso con compromiso visceral; además, son frecuentes la reactivación de formas latentes y la asociación con el curso crónico, las recidivas y la poca mejoría con la terapia ^26^. En el presente estudio, los tres pacientes presentaron formas cutáneas difusas de la leishmaniasis, uno de ellos, además, con compromiso de mucosa oral y nasal; tenían recuentos de CD4 por debajo de 100 células/mm^3^ y, dos de ellos, infección por *Histoplasma* spp.: uno con histoplasmosis pulmonar y otro con histoplasmosis diseminada progresiva y coinfección en la piel por leishmaniasis e histoplasmosis demostradas por PCR. También, se ha descrito la coinfección de *Leishmania* spp. e *Histoplasma* spp., aunque con baja prevalencia y presentaciones atípicas ^27^.

Hasta donde se sabe, este es el primer estudio en Colombia en que se evalúan las manifestaciones dermatológicas de pacientes hospitalizados con HIV y su relación con los recuentos de CD4. Se incluyó una muestra importante de pacientes y se tuvo en cuenta una gran cantidad de variables clínicas y sociodemográficas, lo que permitió caracterizar mejor esta población. Entre las limitaciones del estudio, cabe mencionar su naturaleza retrospectiva, en tanto que el uso de una fuente secundaria de información dificultó la recolección de datos sobre ciertas variables sociodemográficas, como la orientación sexual, el consumo de sustancias psicoactivas y el grado de escolaridad. Tampoco se incluyeron los datos sobre la terapia antirretroviral relativos a los esquemas terapéuticos usados, el tiempo de tratamiento y el cumplimiento, ni a la carga viral por no estar estandarizada en los reportes.

Se concluye que las manifestaciones dermatológicas en pacientes con HIV son motivo frecuente de hospitalización o interconsulta y que estas suelen ser más de una. Cabe resaltar que las manifestaciones infecciosas, incluso las que son definitorias de sida, siguen estando entre las principales causas de hospitalización en países en vía de desarrollo, aunque también se presentan las reacciones a medicamentos con gran frecuencia, siendo las más frecuentes las exantemáticas relacionadas con antibióticos (sulfas).

En cuanto al recuento de linfocitos T CD4, se encontró que era menor de 200 células/mm^3^ en la gran mayoría de los pacientes y que un porcentaje significativo de ellos tenía condiciones desfavorables como desempleo, condición de calle o privación de la libertad, lo que debe tenerse en cuenta en las estrategias de salud pública dirigidas a mejorar su atención integral, de manera que se ajusten a los objetivos de desarrollo sostenible planteados por la Organización de las Naciones Unidas.

Por último, se logró establecer una relación estadísticamente significativa entre el herpes zóster diseminado y los recuentos de CD4 entre 200 y 499 células/mm^3^, lo que sugiere que, aunque estos no sean tan bajos, pueden presentarse formas graves de la enfermedad debido a una posible disfunción de las células T y el agotamiento del sistema inmunológico. Se encontró, asimismo, una relación entre la candidiasis oral y recuentos de CD4 menores de 100 células/mm^3^, lo que se ha reportado en otros estudios y pone de presente el papel de la candidiasis oral como un marcador importante de debilitamiento del estado inmunológico de los pacientes con HIV.
